# An extensive assessment of network alignment algorithms for comparison of brain connectomes

**DOI:** 10.1186/s12859-017-1635-7

**Published:** 2017-06-06

**Authors:** Marianna Milano, Pietro Hiram Guzzi, Olga Tymofieva, Duan Xu, Christofer Hess, Pierangelo Veltri, Mario Cannataro

**Affiliations:** 1 Department of Surgical and Medical Sciences, University of Catanzaro, Catanzaro, Italy; 2 Department of Radiology University of California, San Francisco, USA

**Keywords:** Human connectome, Graph theory, Alignment network algorithms

## Abstract

**Background:**

Recently the study of the complex system of connections in neural systems, i.e. the connectome, has gained a central role in neurosciences. The modeling and analysis of connectomes are therefore a growing area. Here we focus on the representation of connectomes by using graph theory formalisms. Macroscopic human brain connectomes are usually derived from neuroimages; the analyzed brains are co-registered in the image domain and brought to a common anatomical space. An atlas is then applied in order to define anatomically meaningful regions that will serve as the nodes of the network - this process is referred to as parcellation. The atlas-based parcellations present some known limitations in cases of early brain development and abnormal anatomy. Consequently, it has been recently proposed to perform atlas-free random brain parcellation into nodes and align brains in the network space instead of the anatomical image space, as a way to deal with the unknown correspondences of the parcels. Such process requires modeling of the brain using graph theory and the subsequent comparison of the structure of graphs. The latter step may be modeled as a network alignment (NA) problem.

**Results:**

In this work, we first define the problem formally, then we test six existing state of the art of network aligners on diffusion MRI-derived brain networks. We compare the performances of algorithms by assessing six topological measures. We also evaluated the robustness of algorithms to alterations of the dataset.

**Conclusion:**

The results confirm that NA algorithms may be applied in cases of atlas-free parcellation for a fully network-driven comparison of connectomes. The analysis shows MAGNA++ is the best global alignment algorithm. The paper presented a new analysis methodology that uses network alignment for validating atlas-free parcellation brain connectomes. The methodology has been experimented on several brain datasets.

## Background

The brain is a complex organ of vertebrates and it is composed of single specialized cells called neurons. Neurons are connected among them by synapses forming a complex network of connections. Connections among neurons carry signal pulses that carry information [1]. The activity of the brain is mostly due to this set of connections.

Recent studies have demonstrated in an independent way a strict relation among the set of connections, the functions of the brains and the relations among the insurgence of neurological diseases and the variations of mechanims of connections with respect to healthy people [2]. For example, in the Alzheimer Disease a decreased connectivity, and hippocampus changes are detected [3], the Parkinson disease is associated to altered connectivity [3], or in anxiety disorder an increased connectivity and amygdala changes is found [4].

Consequently, the interest for the modeling and the analysis of the whole system of the brain elements and their relations has lead to the introduction of the so-called connectomics, i.e the study of **connectome** referred to as the set of elements and interactions [5]. Connectomics is based on modern technologies of investigation of the brain that are able to take a sort of picture of the brain connections of patients [6]. Connectome may be analyzed using different zoom, e.g. by focusing on single components, i.e. neurons and axons, or grouping them into regions. Usually the analysis of single components is defined to as anatomic connectivity, while the analysis of regions is called functional connectivity because regions are in general perfoming different functions.

**Fig. 1:**
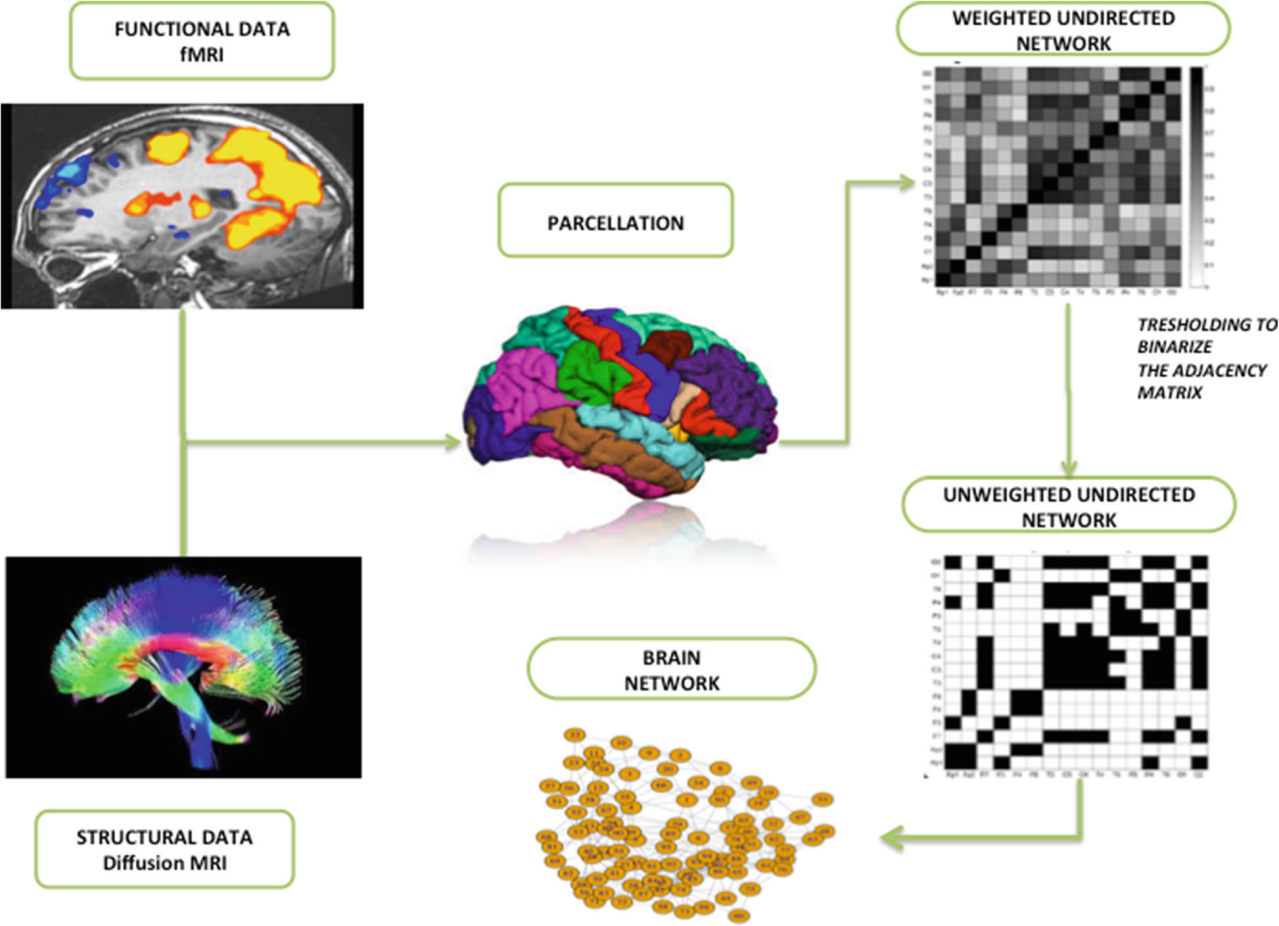
Building a representative network from experimental data: example of a workflow. Diffusion or functional MRI images are acquired for a subject according to the study to be conducted. The MRIs are used to perform whole-brain parcellation by selecting a suitable method. Starting from the parcelled whole brain the computation of connections is performed and a weighted adjacency matrix is constructed. Then, the weights of adjacency matrix are binarized. Finally, the resulting brain network is obtained

Among the others, one of the main sources for deriving information about connectomes is Magnetic Resonance Imaging (MRI) [7]. A typical MRI experiment produces a set of images providing both anatomical and functional information. The first one is constituted by axonal fibers between cortical regions, the second one provides information about the functional connectivity, i.e. the activation of region of interest (ROI). Such analysis is often conducted by using diffusion tensor imaging (DTI) that is a specialised version of Diffusion-weighted magnetic resonance imaging (DWI or DW-MRI), and a DTI has been used extensively to map white matter tractography in the brain through the analysis of patterns of diffusion of molecules through bundles of neural axons. The anatomical connectivity structures are primarily derived through applying tractography algorithms to DTI data. Functional connectivity data are derived from functional magnetic resonance imaging (fMRI). The fMRI images show active regions of the brain at a given instance, based on the blood oxygen consumption level. The obtained networks are called functional networks. The combined use of these two techniques is used to determine the structure of human brain connectome as depicted in Fig. 1.

Once obtained, connectome data needs to be integrated into a suitable model. One of the most used representation of such data is given by the graph theory, whose models have been used by different approaches to extract clinically relevant information [8, 9]. Graph theory ensures the possibility of modeling such data into a single network model and then the possibility to summarize all the characteristics into few measures, giving the understanding of the organization of the entire network as well as individual network elements [10].

Differently to other kind of networks, the modeling of connectomes using graphs is a open research area since there are many possibility for defining the nodes, the edges, that corresponds to different scale of views. For instance, nodes may represent neurons and edges their axons [11]. Here we focus on the representation of region of interest (ROI) as nodes, and the representation of functional or anatomical connections as edges. There exist three main categories of research applied on such networks: (i) the improvement of the reconstruction of graphs starting from MRI images, (ii) the identification of the structure of networks (i.e. which is the theoretical model underlying the brain network organization), (iii) the identification of relevant modules that may be used to understand brain functions and their modifications in case of disease (e.g. for early detection of diseases). The first and the third problem strictly rely on the definition of a framework for the comparison of graphs.

Considering, for instance, the first problem it should be noted that each MRI experiment produces a series of images (either from intra-subject or inter-subject) that need to be aligned into a spatial domain. When using both functional and structural images, coregistration is the process of the alignment of functional and structural images to map functional information into anatomical space. In such a way each region will correspond to a node of a network using an atlas to define anatomically meaningful regions [12].

Nevertheless, such an approach may lead to substantial inaccuracies in cases of abnormal anatomy (e.g. in presence of diseases) and early brain development (e.g. in brain of child). To address this problem, it has been recently proposed to use atlas-free parcellation and to construct and compare individual connectomes only in the network space [13]. In [13] the authors perform the atlas-free parcellation as the finest parcellation that still interconnects the whole brain, leaving no nodes isolated. Then, they group subjects into homogeneous groups and the NA is performed within each group. The sum network is obtained and mapped to the anatomy of a “reference brain.”

Such work, demonstrates the possibility to use NA into the atlas-free parcellation workflow and it poses to the research community the challenge to systematically explore the performance of different NA algorithms since different NA approaches are widely applied in molecular biology analysis, but they have not been explored yet in relation to MRI connectomics.

The techniques for the alignment of biological networks fall into two categories: (i) the local network alignment searches relatively small similar subnetworks that are likely to represent conserved functional structures, (ii) the global network alignment looks for the best superimposition of the whole input networks. However, these approaches can not be easily applied in the connectome alignment problem. The reason is related to the strategy underlying methodology of alignment. For example, the local network aligners, widely used to build the alignment of protein interaction networks (PINs) [14], take as input two networks and a list of seed nodes used to build the initial alignment graph (see [15] for complete details about the construction of the alignment graph). These initial nodes are selected based on biological consideration, such as homology relationships between nodes of PINs. Since the nodes of the brain networks represent ROIs, the homology information cannot be obtained in the case of connectome networks and then, the local alignment cannot be applied.

In this paper we selected six existing state of the art global alignment algorithms and we tested these aligners on diffusion MRI-derived brain networks. The algorithms tested here are MAGNA++ [16], NETAL [17], GHOST [18], GEDEVO [19], WAVE [20], Natalie2.0 [21]. The algorithms are applied to build the alignments among the diffusion MRI-derived brain networks. After the alignments were built, we compared the performance of these algorithms and evaluated this robustness.

### Brain parcellation

**Fig. 2:**
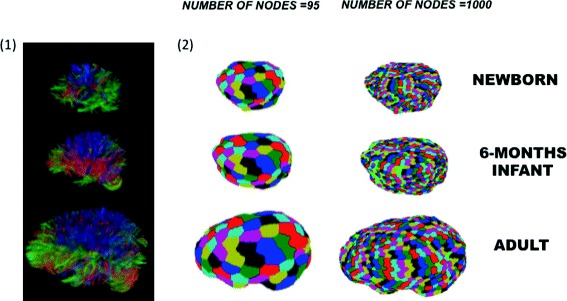
Definition of (*1*) edges and (*2*) nodes using an atlas-free random parcellation and using diffusion MRI and tractography. In the first box the edges reconstruction is reported, whereas in the second box the two kind of whole brain parcellations in newborns, 6 month-old subjects and adults are shown. The first cortical parcellation is performed by setting the number of equal-area nodes equal to 95. The second cortical parcellation is performed by setting the number of equal-area nodes equal to 1000. In this last one it is possible to note disconnected regions highlighted in *green*

An essential step in the analysis and macroscopic mapping of brain network is the subdivision of the brain into large-scale regions, also known as “parcellation process”. The brain parcellation consists of dividing the brain into a set of macroscopic, homogeneous and non-overlapping regions with respect to information provided, generally, by techniques based on magnetic resonance imaging (MRI) [22]. Especially, MRI has allowed to obtain information about anatomical connectivity, functional connectivity, or task-related activation. Different pieces of evidence demonstrate that parcellation of the brain into the homogeneous region is far from being defined, as well as the edges definition and their placement. In the graph representation of a parcellation-based connectome, the nodes of the graph correspond to a brain region and the edges correspond to structural or functional connections between these regions. Despite its relative simplicity, the application of graph theory to the study of connectomes presents some particular challenges related to the meaningful definition of nodes and edges. An ideal model should represent the true subsystems (as nodes) and the true relations (as edges). However, as deeply investigated in [23], there is no clear evidence for the optimal definition of both nodes and edges. For example, an ideal node definition should group a set of neurons to maximize the functional homogeneity within and to maximize the functional heterogeneity among different nodes. Moreover, it should take into account the spatial (and hopefully temporal) relationship among nodes. Besides the definition, the edges representation is also currently an open challenge and this task is related to the type of measured connectivity, and the method used to quantify it. As mentioned above, brain connectivity can refer to different aspects of brain organization including (i) *anatomical connectivity* consisting of axonal fibers connecting cortical and subcortical regions inferred from diffusion imaging (see Fig. 2 (1)), and (ii) *functional connectivity* defined as the observed statistical correlations of the Blood oxygenation level dependent (BOLD) signal between brain regions.

That is, the choice of parcellation scheme has a significant impact on the subsequent analysis. There currently exist three parcellation-based connectome approaches:

1. Parcellation of the brain by using **predefined anatomical templates**. This approach consists of the registration of the structural images from MRI to anatomical atlas based on the Brodmann areas [24]. In this way, the whole brain is subdivided into labeled regions according to the different labels regions of the templates;
2. Parcellation of the brain by using **randomly generated templates** [25]. For the random parcellation different algorithms are applied to produce parcels of roughly equal size. Thus, the generated templates are characterized by approximately uniformly sized brain regions to avoid anatomical biases;
3. **Connectivity-based parcellations** that aim to delineate brain regions by analyzing the similarities in structural or functional connectivity patterns. Based on the notion that regions with a similar connectivity profile are involved in the same analogous functional roles, the connectivity-based parcellation partitions small seed regions into a largest collection of functionally homogeneous brain regions by clustering seeds with similar connectivity profiles.

However, each method presents some pitfalls. For example, the registration of brain of the studied subject to a generic brain with defined Brodmann areas raises the question of the accuracy of mapping. In fact, in the most of the cases, the borders of the Brodmann areas, originally defined using cytoarchitectural differences between brain regions, do not match with the cortical surface analyzed.

This approach is limited by inter-subject variability and can be especially problematic in the context of brain maturation. Furthermore, it has been demonstrated that parcellation of brain with predefined anatomical templates may impact negatively all the subsequent analysis by introducing evident biases [13]. In this paper we focus on the random, atlas-free definition of nodes in individual subjects (see Fig. 2 (2)), which can allow for a fully network-driven study of the brain and for comparing brains of different subjects and, potentially, species [13].

### Global network alignment algorithms

The identification of an accurate node mapping between atlas-free networks may offer significant details on the comparison of brains or structure of groups of subjects, such as healthy versus diseased subjects. Many different network alignment methods have been proposed in biological fields [26].

Formally, a graph G is defined as *G*={*V*,*E*}, where V is a finite set of nodes and E is a finite set of edges. Let *G*_1_={*V*_1_,*E*_1_} and *G*_2_={*V*_2_,*E*_2_} be two graphs, where *V*_1,2_ are sets of nodes and *E*_1,2_ are sets of edges, a graph alignment is the mapping between the nodes of the input networks that maximizes the similarity between mapped entities. From a theoretical point of view, the graph alignment problem consists of finding an alignment function (or a mapping) *f*:*V*_1_→*V*_2_ that maximizes a cost function *Q*. The similarity between the graphs is defined by a cost function, *Q*(*G*_1_,*G*_2_,*f*), also known as the quality of the alignment.

Let *f* be an alignment between two graphs *G*_1_ and *G*_2_, given a node *u* from *G*_1_, *f*(*u*) is the set of nodes from *G*_2_ that are aligned under *f* to *u*. Q expresses the similarity among two input graphs with respect to a specific alignment *f* and the formulation of Q strongly influences the mapping strategy.

There exist different formulations of Q that fall into following the classes:

**Topological Similarity**: Graphs are aligned by considering only edge topology, so that the perfect alignment is reached when input graphs are isomorphic.

Usually, the cost function is defined as the number of edges conserved by *f* with respect to the total number of edges in the source network (*G*_1_), also referred to as edge correctness (EC) [27]. Therefore, the EC does not take into account the target network (*G*_2_).

1 $$ EC= \frac{(v_{1},v_{2})\in E_{1}| f(v_{1},v_{2})|\in E_{2} }{|E_{1}|}  $$

Another typical measure is the Induced Conserved Structure, *ICS* [27]. Let *D* be the number of edges in a subnetwork of *G*_2_ induced on the nodes in *G*_2_ aligned to the nodes in *G*_1_, ICS of *f* is the ratio of the number of edges conserved by *f* to *D*.

2 $$ ICS= \frac{|f(E_{1})| }{|E(G_{2}[f(V_{1})])|}  $$

where *D* is |*E*(*G*_2_[*f*(*V*_1_)])|.

However, ICS fails in the penalization of misaligning edges in the smaller network because it takes into account the target network.

Finally, the Symmetric Substructure Score, *S* ^3^[27], takes into account the unique edges in the composite graph created by the overlap of two networks.

3 $$ S^{3}= \frac{|f(E_{1})| }{|E_{1}|+|E(G_{2}[f(V_{1})])|-|f(E_{1})|}  $$

*S* ^3^ has been shown to be superior to existing measures since it penalizes both alignments from sparse graph regions to dense graph regions and alignments from dense graph regions to sparse graph regions.

**Node Similarity**: Such function considers the similarity among mapped nodes. Nodes of the aligned graphs can be more or less similar to each other. Thus the alignment should align each node of one graph to the most similar node of the other one given a node similarity functions, *s*(*v*_1_,*v*_2_)→*R*, *v*_1_∈*V*_1_, *v*_2_∈*V*_2_. The overall objective is to maximize the sum of scores considering aligned nodes.

4 $$ NC=max {sum}_{v_{1},v_{2}}=f(v_{1})s(v_{1},v_{2})  $$

**Hybrid approaches**: Some recent formulations of Q take into account of both of the approaches by linear combination.

The network alignment problem can be formulated in various ways. In general, the network alignment can be classified as local alignment or global alignment.

The *local alignment* aims to find multiple and unrelated regions of isomorphism, i.e. same graph structure, between the input networks, where each region implies a mapping independently of other regions. The strategy consists of the mapping or set of mappings between subsets of nodes such that their similarity is maximal over all possible subsets. These subnetworks correspond to conserved patterns of interaction that can represent a conserved motif or pattern of activities (a synopsis is available in [15]). The *global alignment* aims to find a mapping that should cover all of the nodes of the input networks, associating each node of a network with one node of the other networks or marking the node as a gap when no possible match exists. This strategy does not consider small regions of similarity, i.e. conserved motifs, but tries to find a consistent mapping between the whole set of nodes of the networks.

In this work, six global alignment algorithms were chosen to built the global alignment of brain networks. We give hereafter a short conceptual description.

A popular existing method of global alignment is MAGNA [16]. MAGNA is a global network aligner that simulates a population of alignments that evolves over time by applying a genetic algorithm and a function for the crossover of two alignments into a superior alignment. Since the genetic algorithm simulates the evolutionary process guided by the survival of the fittest principle, only alignments, i.e. those that conserve the most edges, survive. Thus, MAGNA proceeds to the next generation, until the alignment accuracy cannot be optimized further. Recently, an extension of MAGNA algorithm called MAGNA++ was developed.

The second aligner is NETAL [17], an algorithm for the global alignment widely used to protein-protein interaction networks. NETAL builds the best global network alignment by applying a greedy method, based on the alignment scoring matrix, which is derived from both biological and topological information of input networks.

The third algorithm, GHOST [18], is a global pairwise network aligner that uses a novel spectral signature based on the local neighborhood’s topology to measure topological similarity between subnetworks. The idea behind GHOST consists of the combination of the novel novel spectral signature with seed-and-extend procedure to build the alignment.

The fourth global aligner is GEDEVO [19], a novel tool for efficient graph alignment.

Underlying the GEDEVO method is the Graph Edit Distance model (GED), where a graph is transferred into another one with a minimal number of edge insertions and deletions. Thus, GEDEVO uses the GED as optimization model for finding the best alignments.

The fifth algorithm is WAVE [20] a general and novel alignment strategy which aim is to optimize both node and edge conservation while constructing an alignment. WAVE is used on top of an established node cost function and it leads to a new superior method for global network alignment, by favoring conserved edges among nodes with node cost function similar over those with node cost function dissimilar.

The last algorithm is Natalie2.0 [21], a network alignment method, which looks at the network alignment problem as a generalization of the quadratic assignment problem and solves it using techniques from integer linear programming.

## Results and discussion

### Dataset

The dataset consisted of 24 diffusion MRI-derived structural networks of human brain: 12 networks with a number of nodes equal to 95 and the 12 networks with a number of nodes equal to 1000. The brain networks are related to three different stages of development by including newborns (NE), six-month-old infants (6M), and adults (AD). See *Methods* *Section* for a complete description.

### Building of brain network alignment

We built the alignment of all networks with 95 and 1000 nodes (for convenience we call the two dataset *n* *e* *t* *w* *o* *r* *k* *s*_95_ and *n* *e* *t* *w* *o* *r* *k* *s*_1000_) related to same growth stages (NE, 6M, AD) by applying MAGNA++, NETAL, GHOST, GEDEVO, WAVE and Natalie2.0 algorithms. Initially, we aligned each network with itself. We executed this stage in order to test if the algorithm is able to build the alignment (see [28] for more details). Then, we aligned the brain network related to the same growth stage, NE, 6M, AD. We run all NA methods on the same Linux machine with Intel Core i5 and 4GB of RAM. We also generated the same alignments using the six NA algorithm selected. We selected the following MAGNA++ parameters: *S* ^3^ as measure of Edge Conservation, the *α* parameter equal to 0, in order to consider only topology, whereas the population size, number of generation, fraction of elite members were set to default values. We tested different parameters and obtained best results with the default parameters for NETAL, GHOST, GEDEVO, Natalie2.0. WAVE did not require to set specific parameters. The NETAL parameters were: *a* that controls the weight of similarity and interaction scores, *b* that controls the weight of biological and topological similarities, *c* that controls the contribution of neighbors of two nodes in calculating the similarity between them, *i* that defines the number of iterations for computing similarities. In GEDEVO, *pop* parameter that controls the number of new individuals per iteration set equal to 1000 and *maxsame* that controls the stop after N iterations without any score improvement were equal to 300. In Natalie2.0, *beta* set equal to 1, in order to consider only topology, whereas, *maxJsonNodes* that controls maximum number of nodes to be generated and *verbosity* that specifies the verbosity level parameters were set to default values. To build the alignment using GHOST, *nneighbors* was set to all, *serchiter* that controls the number of local search iterations that should be performed after the initial global alignment is complete, set equal to 10, *beta* that controls the edges alignment in the initial seed-and-extend phase of the algorithm, set equal to 1, *ratio* that controls ratio of bad-moves allowed during the local-search phase of the alignment algorithm, set equal to 8.0.

The global alignments were built among the *n* *e* *t* *w* *o* *r* *k* *s*_95_ and then among the *n* *e* *t* *w* *o* *r* *k* *s*_1000_.

At the end of this alignment step, we built 48 global alignments for each selected aligner by using the dataset *n* *e* *t* *w* *o* *r* *k* *s*_95_. Table 1 presents all the obtained alignments.

**Table 1:** List of the alignments built among the networks with 95 nodes

Network	Pairwise alignments
NE01	NE01 vs NE01; NE01 vs NE02; NE01 vs NE03; NE01 vs NE04
NE02	NE02 vs NE01; NE02 vs NE02; NE02 vs NE03; NE02 vs NE04
NE03	NE03 vs NE01; NE03 vs NE02; NE03 vs NE03; NE03 vs NE04
NE04	NE04 vs NE01; NE04 vs NE02; NE04 vs NE03; NE04 vs NE04
6M01	6M01 vs 6M01; 6M01 vs 6M02; 6M01 vs 6M03; 6M01 vs 6M04
6M02	6M02 vs 6M01; 6M02 vs 6M02; 6M02 vs 6M03; 6M02 vs 6M04
6M03	6M03 vs 6M01; 6M03 vs 6M02; 6M03 vs 6M03; 6M03 vs 6M04
6M04	6M04 vs 6M01; 6M04 vs 6M02; 6M04 vs 6M03; 6M04 vs 6M04
AD1	AD1 vs AD1; AD1 vs AD2; AD1 vs AD3; AD1 vs AD4
AD2	AD2 vs AD1; AD2 vs AD2; AD2 vs AD3; AD2 vs AD4
AD3	AD3 vs AD1; AD3 vs AD2; AD3 vs AD3; AD3 vs AD4
AD4	AD4vs AD1; AD4 vs AD2; AD4 vs AD3; AD4 vs AD4

About the *n* *e* *t* *w* *o* *r* *k* *s*_1000_, we built 48 alignments with NETAL, GHOST, GEDEVO, WAVE according to Table 1. Since MAGNA++ requires that network 1 has fewer nodes than network 2 to build the global alignment, we aligned each smaller network, in term number of nodes, to larger networks. Finally we obtained 30 alignments built with MAGNA++. We do not have alignments by using Natalie2.0 because the algorithm was not able to build the alignment among networks with high nodes number.

Table 2 reports the execution time to build the alignment on the networks with 95 nodes and on the networks with 1000 nodes for all global alignment algorithms.

**Table 2:** Execution Time to build the global alignment with MAGNA++, NETAL, GHOST, GEDEVO, WAVE, Natalie2.0 for the networks with 95 nodes and the networks with 1000 nodes

	Execution time for network	Execution time for network
	with 95 nodes	with 95 nodes
MAGNA++	1200 seconds	1800 seconds
NETAL	2 seconds	4 seconds
GHOST	2 seconds	10 seconds
GEDEVO	120 seconds	0.2 seconds
WAVE	5 seconds	5 seconds
Natalie2.0	60 seconds	-

### Topological evaluation

Here, we aim to evaluate the quality of the alignments built with MAGNA++, NETAL, GHOST, GEDEVO, WAVE, Natalie2.0 NA algorithms. The topological quality is related to two alignment algorithm capability as the reconstruction of the true node mapping and the conservation of as much as possible edges. Typically, the Node Correctness (NC) is the measure widely used to evaluate how an alignment reconstructs the true node mapping correctly. Instead, different measures are used to evaluate how well the edges are conserved on an alignment, such as EC, ICS or *S* ^3^ (see the previous Section). However, among the selected algorithm, MAGNA++ is the unique tool that enables to compute all quality measures, NC, EC, ICS and *S* ^3^. For this reason, we computed the quality of built alignments by using the software for NA evaluation proposed in [26]. The software ensures the computation of six topological measures: Precision Node Correctness (P-NC), Recall Node Correctness (R-NC), F-score of Node Correctness (F-NC), High Node coverage (NCV), Generalized *S* ^3^ (G *S* ^3^), and NCV combined with G *S* ^3^ (NCV-G *S* ^3^). P-NC evaluates the the precision of the alignment i.e. the percentage of the aligned node pairs that are also present in the true node mapping. P-NC is defined as:

5 $$ P-NC= \frac{(M \cap N)}{(M)}  $$

were M is the set of node pairs that are mapped under the true node mapping and N be the set of node pairs that are aligned under *f*.

R-NC evaluates the percentage of all node pairs from the true node mapping that are aligned under *f* and it is defined as:

6 $$ R-NC= \frac{(M \cap N)}{(N)}  $$

G *S* ^3^ is the percentage of conserved edges *N*_*c*_ out of the total of both conserved and non-conserved edges *N*_*n*_:

7 $$ GS^{3}= \frac{N_{c}}{N_{c}+N_{n}}  $$

NCV is the percentage of nodes from G1 and G2 that are also in G’1 and G’2 subgraphs:

8 $$ NCV= \frac{V'_{1}+V'_{2}}{V_{1}+V_{2}}  $$

Finally, NCV-G *S* ^3^ is the geometric mean of the NCV and G *S* ^3^ measures. These six measures evaluate alignment quality from different aspects and they can be divided in two groups, the first one composed by P-NC, R-NC and F-N measures that estimate how well the alignment captures the true node mapping, and the second one formed by NCV, G *S* ^3^ and NCV-G *S* ^3^ measures that capture the size of the alignment. We computed P-NC, R-NC, F-NC, NCV, G *S* ^3^ and NCV-G *S* ^3^ for each alignment built with MAGNA++, NETAL, GHOST, GEDEVO, WAVE, Natalie2.0. Then, we compared these measures in order to analyze which algorithm ensures a higher alignment quality. However, we focus on F-NC and NCV-GS3 as the most representative non-redundant measures because these are both a combination of two individual measures.

**Fig. 3:**
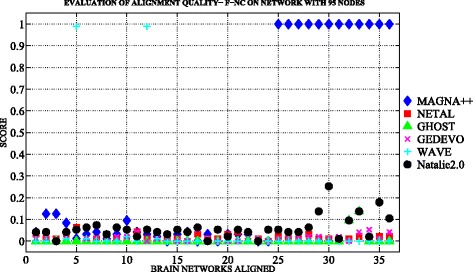
The topological evaluation of alignments built with MAGNA++ (*blue marker*), NETAL (*red marker*), GHOST (*green marker*), GEDEVO (*purple marker*), WAVE (*light blue marker*), Natalie2.0 (*black marker*). The Figure shows the F-NC scores of each alignment built among the networks with 95 nodes by applying the selected six aligners

**Fig. 4:**
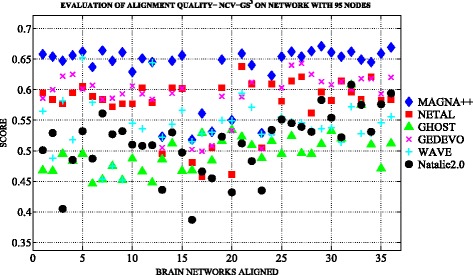
The topological evaluation of alignments built with MAGNA++ (*blue marker*), NETAL (*red marker*), GHOST (*green marker*), GEDEVO (*purple marker*), WAVE (*light blue marker*), Natalie2.0 (*black marker*). The Figure shows the NCV-G *S* ^3^ scores of each alignment built among the networks with 95 nodes by applying the selected six aligners

**Fig. 5:**
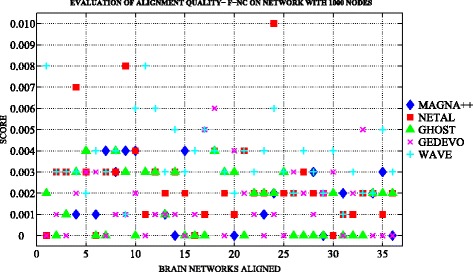
The topological evaluation of alignments built with MAGNA++ (*blue marker*), NETAL (*red marker*), GHOST (*green marker*), GEDEVO (*purple marker*), WAVE (*light blue marker*), Natalie2.0 (*black marker*). The Figure shows the F-NC scores of each alignment built among the networks with 1000 nodes by applying the selected six aligners

**Fig. 6:**
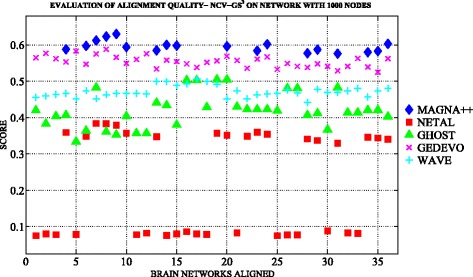
The topological evaluation of alignments built with MAGNA++ (*blue marker*), NETAL (*red marker*), GHOST (*green marker*), GEDEVO (*purple marker*), WAVE (*light blue marker*), Natalie2.0 (*black marker*). The Figure shows the NCV-G *S* ^3^ scores of each alignment built among the networks with 1000 nodes by applying the selected six aligners

Figures 3 and 4 show an overview of topological measures comparison on *n* *e* *t* *w* *o* *r* *k* *s*_95_ whereas Figs. 5 and 6 show an overview of topological measures comparison on *n* *e* *t* *w* *o* *r* *k* *s*_1000_.

We note that the best results in terms of edge conservation were obtained when applying MAGNA++ as global aligner both on *n* *e* *t* *w* *o* *r* *k* *s*_95_ and *n* *e* *t* *w* *o* *r* *k* *s*_1000_. We also note that values of NCV-G *S* ^3^ for *n* *e* *t* *w* *o* *r* *k* *s*_95_ are higher than NCV-G *S* ^3^ for *n* *e* *t* *w* *o* *r* *k* *s*_1000_.

Regarding the reconstruction of the true node mapping we note that the quality of alignment is homogeneous among *n* *e* *t* *w* *o* *r* *k* *s*_95_, with exception of the quality of 12 alignments built with MAGNA++ that was better than other algorithms. For the *n* *e* *t* *w* *o* *r* *k* *s*_1000_, the F-NC values are higher for the alignment built with WAVE with exception of the alignments built with NETAL.

### Robustness analysis

**Fig. 7:**
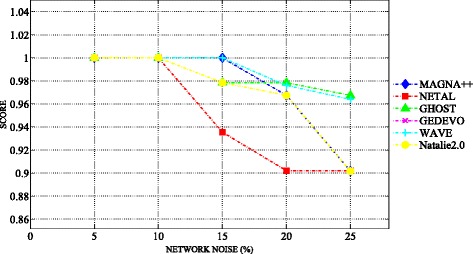
The robustness evaluation of alignments to various alteration levels of networks with 95 nodes. The figure shows the trend of the edge conservation related to alignment of the high-confidence brain network with the synthetic counterparts at 5, 10, 15, 20 and 25% of added noise. The alignments are built with MAGNA++ (*blue marker*), NETAL (*red marker*), GHOST (*green marker*), GEDEVO (*purple marker*), WAVE (*light blue marker*), Natalie2.0 (*black marker*)

**Fig. 8:**
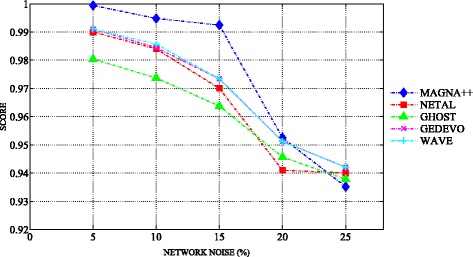
The robustness evaluation of alignments to various alteration levels of networks with 1000 nodes. The figure shows the trend of the edge conservation related to alignment of the high-confidence brain network with the synthetic counterparts at 5, 10, 15, 20 and 25% of added noise. The alignments are built with MAGNA++ (*blue marker*), NETAL (*red marker*), GHOST (*green marker*), GEDEVO (*purple marker*), WAVE (*light blue marker*), Natalie2.0 (*black marker*)

We analyzed the robustness of the different algorithms to various levels of graph alteration (edge removal). We generated a series of altered networks derived from the high-confidence brain network. We built the synthetic counterparts with 5, 10, 15, 20 and 25% of added noise. We obtained 60 synthetic networks with 95 nodes and 60 synthetic networks with 1000 nodes. To measure the performance of MAGNA++, NETAL, GHOST, GEDEVO, WAVE, Natalie2.0, we aligned the high-confidence brain network with its noisy counterparts obtained by random removal of edges from the network. Since all networks contain the same nodes, we know the true node mapping. The high-confidence network is an exact subgraph of each noisy network. Exploiting randomness, we ran each experiment 30 times and averaged results over the 30 runs [26]. The aim of the test was to demonstrate that the alignment algorithms are capable of producing high-quality alignments with edge conservation of about 90%. This evaluation test has been widely adopted in different NA studies (see [18, 29]). We performed this test on the brain networks built with each selected global aligner and with NETAL. The results show that, given the high topological similarity of the aligned network with its noisy counterpart, MAGNA++, NETAL, GHOST, GEDEVO, WAVE, Natalie2.0 are capable of discovering alignments with high edge conservation. The better performance was achieved with MAGNA++. Figures 7 and 8 show the validation of the edge conservation when introducing increasing noise level from 5 to 25% into the high-confidence brain networks.

## Conclusion

Understanding brain connectivity can shed light on the brain cognitive functioning that occurs via the connections and interaction between neurons. The term brain connectivity refers to different aspects of brain organization including anatomical connectivity consisting of axonal fibers across cortical regions and functional connectivity defined as the observed statistical correlations of the BOLD signal between regions of interest. A powerful formalism to represent the brain connectivity derives from graph theory. The graph theoretical modeling of the human connectome has already enabled important discoveries and will most likely continue to do this in the future. In this study we proposed to apply classical global alignment algorithms such as MAGNA++, NETAL, GHOST, GEDEVO, WAVE, Natalie2.0, to align atlas-free human brain networks at three developmental stages. We analyzed the alignment results in term of topological quality measures and performance. According to these analyses, MAGNA++ resulted the best alignment algorithm. Our ongoing study is focused on the implementation of an ad hoc algorithm for connectome alignment. Since there are many conditions in which the classical parcellation is not useful, we retain that this seminal work may open the way for the use of network alignment in atlas-free parcellation.

## Methods

### Data set

The dataset consisted of diffusion MRI-derived structural networks of human brain at different stages of development, starting with newborns [13]. Acquisition of the MRI data was compliant with the Health Insurance Portability and Accountability Act (HIPAA) and the study was approved by the Committee on Human Research (CHR) of the University of California, San Francisco. Three age groups were included: 4 newborns imaged in the first 4-5 days of life (NE), 4 six-month-old infants (6M), and 4 adults (age 24-31 years) (AD). The two pediatric groups had transient encephalopathy at birth, but none of the patients had clinical or imaging evidence of brain injury. The subjects were scanned on a 3T GE MR scanner using a spin echo (SE) echo planar imaging (EPI) diffusion tensor imaging DTI sequence with parameter described in [13]. Tensor calculation, tractography, cortical parcellation into 95 equal-area nodes and then in 1000 equal-area nodes (Fig. 2), and construction of the connectivity matrices was performed as described previously [13]. All networks were binarized with a threshold of 1 streamline. Starting form the images we obtained two different datasets. The first dataset consist of 12 networks with number of nodes equal to 95 depending on parcellation step. For convenience we call this dataset *n* *e* *t* *w* *o* *r* *k* *s*_95_. Table 3 shows the networks parameters. About the second dataset, the 12 networks were constructed by setting the number of equal-area nodes for the cortical parcellation equal to 1000. Since all cortical areas of the brain are connected, a fine parcellation should ensure the interconnectedness of the whole brain, leaving no nodes isolated. In [13] the authors demonstrated that the highest number of nodes at which this condition is fulfilled in equal to 95. For this reason, the networks of the second dataset showed the isolated nodes that were not computed in the construction of the connectivity matrices. For convenience we call this dataset *n* *e* *t* *w* *o* *r* *k* *s*_1000_ even though the nodes number is different from 1000. Table 4 shows the network parameters.

**Table 3:** Details of brain networks with 95 nodes used for experiments

Network	Nodes	Edge	Clustering coefficient	Connected components	Characteristic path length	Density
NE01	95	341	0,394	1	2952	0,079
NE02	95	341	0,376	1	3092	0,075
NE03	95	334	0,364	1	3172	0,072
NE04	95	320	0,394	1	2968	0,076
6M01	95	353	0,42	1	3129	0,076
6M02	95	333	0,429	1	3,027	0,08
6M03	95	333	0,383	1	3,16	0,075
6M04	95	338	0,406	1	3,289	0,072
AD1	95	449	0,445	1	2,723	0,101
AD2	95	406	0,423	1	2,774	0,091
AD3	95	438	0,423	1	2,741	0,098
AD4	95	416	0,42	1	2,742	0,093

**Table 4:** Details of brain networks with 1000 nodes used for experiments

Network	Nodes	Edge	Clustering coefficient	Connected components	Characteristic path length	Density
NE01	889	2555	0	53	11531	0,002
NE02	904	2618	0	71	12255	0,002
NE03	900	2585	0	115	13970	0,002
NE04	899	2298	0	72	11134	0,002
6M01	902	2458	0	47	11446	0,002
6M02	849	2182	0	63	10839	0,002
6M03	805	1928	0	65	13515	0,002
6M04	851	2087	0	63	11444	0,002
AD1	902	3146	0	38	8529	0,002
AD2	869	2691	0	52	8719	0,002
AD3	878	3262	0	58	7918	0,002
AD4	853	2907	0	48	8334	0,002

### Alignment algorithms

In this section we describe in detail the global alignment algorithm selected to align the diffusion brain networks.

MAGNA [27] is a global network aligner that uses a genetic algorithm to build an improved alignment starting from existing ones (generated randomly or by using other aligners). While the alignment is constructed, MAGNA optimizes the edge conservation, without decreasing the quality of node mapping. MAGNA is the first algorithm that uses genetic algorithms to build global alignment. In specific, MAGNA simulates a population of alignments that evolves over time by applying the genetic algorithm and a function for crossover of two alignments into a superior alignment. The genetic algorithm simulates the evolutionary process, guided by the survival of the fittest principle. The genetic algorithm input consist of a initial population of a given number of members. In MAGNA, the members of a population are alignments. Members of a population crossover with each other to produce new members. Only the fittest members are more likely to crossover. Thus, the child resulting from a crossover function reflects each parent. To avoid the size of the population to grow without bound, the size is kept constant across all generations, with only the fittest members surviving from one generation to the next. Thus, as the algorithm progresses, only fittest alignments, i.e. those that conserve the most edges, survive and MAGNA proceeds to the next generation, until the alignment accuracy cannot be optimized further. The fittest alignment from the last generation is reported as the final alignment. Practically, MAGNA takes as input two networks with different nodes number (|*V*_1_|<|*V*2|) and builds the final global alignment. Moreover, to build the alignment MAGNA requires several parameters such as the type of initial population, population size, maximum number of generations (i.e. iterations of the genetic algorithm), and optimization function (i.e., alignment quality measure). Furthermore, MAGNA introduces new and superior alignment quality measure that takes the best from each existing measure, The Symmetric Substructure Score (*S* ^3^) [27]. that takes into account the unique edges in the composite graph created by the overlap of two networks:

9 $$ S^{3}= \frac{|f(E_{1})| }{|E(G_{2}[f(V_{1})-|f(E_{1})| |}  $$

*S* ^3^ has been shown to be superior to existing measure, see [27] for more details.

There exists a MAGNA extension, named MAGNA++ [30], that introduces important improvements.

While MAGNA maximizes edge conservation during the alignment, MAGNA++ enables both the maximization of any different measures of edge conservation (EC, ICS, *S* ^3^) and any desired node conservation measure. Let us define *S*_*N*_ and *S*_*E*_, as node and edge conservation measures, then MAGNA++ maximizes the following measure:

10 $$ \alpha S_{E} + (1-\alpha)S_{N}  $$

where *α* controls the contribution of each node and edge conservation measures and takes the values between 0 and 1. In this way the alignment quality results are improved when MAGNA++ is compared with only node conservation or only edge conservation. Moreover, MAGNA++ provides a graphical user interface for easy use and offers source code for easy extensibility.

NETAL [17] is a global aligner tool that applies a greedy method, based on the alignment scoring matrix derived from biological and topological information of input networks to find the best global network alignment. The alignment building consists of two phases. In the first phase, the Alignment Score Matrix is constructed, exploiting two matrices called Similarity Score Matrix and Interaction Score Matrix. The Similarity Score Matrix is generated from the weighted sum of topological and biological similarities between every two nodes of input networks. The topological and biological similarities are respectively based on the structure of the networks and biological properties (for example in proteins networks). During the building of the alignment the score of the matrix remain always fixed. The Interaction score is based on the estimate of expected value of the number of conserved interactions that involve the node of network 1 aligned with the node of network 2. Since the expected value of the number of conserved interactions changes after the alignment, the interaction scores should be updated iteratively, just as the alignment score matrix should change. In the second phase, a greedy search is used to find the global alignment based on the values of the alignment score matrix. At first, the node pairs with maximum alignment score are chosen and aligned to each other. Then interaction score matrix is updated, and the alignment score matrix is changed based on the new values. The greedy search proceeds until all the nodes of the first network are aligned with the nodes of the second network. Practically, given two networks, NETAL finds an injective mapping so each node in the smaller network is mapped to one node in the larger network. The output consists of final global alignment. Moreover, to build the alignment NETAL requires several parameters such as *a* value that controls the weight of similarity and interaction scores, *b* that controls the weight of biological and topological similarities, *c* that controls the contribution of neighbors of two nodes in calculating the similarity between them, *i* value that defines the number of iterations for computing similarities.

GHOST is global pairwise network aligner widely used in PINs analysis. GHOST introduces a novel spectral signature based on the application of spectral graph theory to build a topological similarity measure. Initially, a set of different spectra and signatures are extracted by considering the induced subgraphs for a range of different radii centered about each node of the network. Then, the structural distance, defined on the spectral densities of two graphs, is computed between the signatures of two nodes of different networks for a sequence of radii. This signature is used to compute the similarity of nodes between different networks and to guide the building of the final global alignment. The alignment is built in two phases. In the fist phase, GHOST applies a seed-and-extend strategy to detect seed regions of an alignment. The seed regions consist of the pairs of nodes from the different networks which the structural distance is minimal. Then, GHOST expands the alignment around the neighborhoods of these pairs of nodes until all nodes of the smaller network are aligned with the nodes of the larger network. In the second phase, GHOST applies a local search strategy to improve the alignment. GHOST explores the pair of nodes aligned and realigns the nodes to obtain an alignment similar to the initial one but with superior topological quality.

GEDEVO is a global network aligner based on an evolutionary algorithm that uses the Graph Edit Distance (GED) as optimization model for finding the best alignments. The GED is a general model for the Graph Matching problem and it is defined as the minimal modifications required to transfer a graph into another graph. So, lets one-to-one mapping *f* among two networks, the GED model counts the inserted or deleted edges induced by the mapping *f*. According to this, the best alignment shows the lowest graph editing cost. The GEDEVO builds the alignment by generating an initial mapping *f* with random permutations and then each pair of nodes is evaluated by using the pairScore. The pairScore reflects how well two nodes correspond in a given mapping *f*. The pairScore depend on: the GED that computes the number of deleted and inserted edges induced by mapping of two nodes given the mapping *f*, and the graphlet signature distance (GSD) that computes the difference in neighboring topologies of two nodes within a distance equal to 4. Afterward, the mapping *f* is partitioned into two sets of pairs with low and hight scores. The high scoring pairs are swapped randomly, whereas a number of randomly chosen bad pairs in the mapping are swap with directed mutations. After each swap, the scores among the new pairs are recomputed. This operation enables to keep good pairs and to swap a bad pair more often with another bad pair. At the end, the one swap that induces the best score is kept. In this way, the final score of the mapping results improved.

Weighted Alignment VotEr (WAVE) is a novel algorithm which builds an alignment by maximizing both node and edge conservation. When WAVE is used on top of well-established node cost functions, the alignment results improved with respect to different methods that optimize only node or edge conservation or treat each conserved edge the same. The reason consists of the capability of WAVE to favor conserved edges with similar NCF end nodes over those with dissimilar NCF end nodes. Furthermore, WAVE introduces a novel measure of edge conservation denoted as weighted edge conservation (WEC). WEC measure counts the number of conserved edges and weights each conserved edge by the node cost function based similarity of its end nodes. Thus, the edges with highly similar end nodes are preferred to be aligned over the edges with dissimilar end nodes. Starting from an empty alignment, WAVE calculates the marginal gain of adding an available node pair to the alignment. The marginal gain depends on the Alignment Quality that is based on a combination of weighted edge conservation and weighted node conservation measures. Thus, the pair with the highest marginal gain is aligned. Furthermore, when a pair of nodes is aligned, this node pair has a chance to give a weighted vote to their neighbors, where weighted vote for the initial vote of each node pair derives from a node conservation measures. At each step WAVE aligns each node pair with the highest vote and votes for all the pairs of neighbors. Finally, the vote that a node pair gets from its aligned neighbors is the marginal gain to the objective function of aligning them.

Natalie 2.0 is an open source software for global network alignment which supports different scoring schemes taking into account both node-to-node correspondences and network topologies. By formulating the global network alignment as a mathematical program, this can be considered as a special case of the well-studied quadratic assignment problem. Natalie 2.0 focuses on sparse network alignment, where each node can be mapped only to a typically small subset of nodes in the other network. This corresponds to a quadratic assignment problem instance with a symmetric and sparse weight matrix. Thus, Natalie 2.0 obtains strong upper and lower bounds for the problem by improving a Lagrangian relaxation approach.

### Assessment of alignment algorithms

To compute the quality of built alignments we used the software for fairly evaluating a NA method proposed by [26]. The software provides a GUI and python source code for any platform. The software aims to analyze an alignment allowing both topological and biological evaluation. In this work we focused only on a topological evaluation. The software requires an input alignment built with any NA method. This input alignment must be provided in the form of aligned node pairs. Once the alignment is supplied, six topological measures, Precision NC (P-NC), Recall NC (R-NC), F-score (F-NC), High Node coverage (NCV), Generalized *S* ^3^ (G *S* ^3^), and NCV combined with G *S* ^3^ (NCV-G *S* ^3^) can be selected. To compute topological evaluation with P-NC, R-NC, and F-NC, the true node mapping between the aligned networks as additional input must be provided. For the topological measures NCV, GS3 and NCV-GS3, the two aligned networks are required as additional input. The biological evaluation is allowed by selecting four measures, GO correctness (GC), Precision of known protein function prediction (P-PF), Recall of known protein function prediction (R-PF) and F-score of known protein function prediction (F-PF). For the calculation of these biological measures, GO data of both aligned networks are required as input. The run time to compute the evaluation measures is few seconds.

## Abbreviations

AD: Adults
BOLD: Blood oxygenation level dependent
CHR: Committee on human research
DTI: Diffusion tensor imaging
DWI: Diffusion weighted imaging
DW-MRI: Diffusion weighted magnetic resonance imaging
EC: Edge correctness
F-NC: F-score
F-PF: F-score of known protein function prediction
fMRI: Functional magnetic resonance imaging
GC: GO correctness
GED: Graph edit distance
GSD: Graphlet signature distance
G *S*^3^: Generalized *S* ^3^
HIPAA: Health insurance portability and accountability act
ICS: Induced conserved structure
MRI: Magnetic resonance imaging
NA: Network alignment
NE: Newborns
NCV: High node coverage
NCV-G *S*^3^: NCV combined with G *S* ^3^
PINs: Protein interaction networks
P-NC: Precision NC
P-PF: Precision of known protein function prediction
ROIs: Region of interest
R-NC: Recall NC
R-PF: Recall of known protein function prediction
*S*^3^: Symmetric substructure score
6M: Six-month-old
WEC: Weighted edge conservation
WAVE: Weighted alignment voter

## References

[1] Kiani R, Cueva CJ, Reppas JB, Peixoto D, Ryu SI, Newsome WT (2015). Natural grouping of neural responses reveals spatially segregated clusters in prearcuate cortex. Neuron.

[2] Bargmann CI, Marder E (2013). From the connectome to brain function. Nat Methods.

[3] Lenka A, Naduthota RM, Jha M, Panda R, Prajapati A, Jhunjhunwala K, Saini J, Yadav R, Bharath RD, Pal PK (2016). Freezing of gait in parkinson’s disease is associated with altered functional brain connectivity. Parkinsonism Relat Disord.

[4] Stein MB, Simmons AN, Feinstein JS, Paulus MP (2007). Increased amygdala and insula activation during emotion processing in anxiety-prone subjects. Am J Psychiatr.

[5] Sporns O, Tononi G, Kötter R (2005). The human connectome: a structural description of the human brain. PLoS Comput Biol.

[6] Xia M, He Y (2011). Magnetic resonance imaging and graph theoretical analysis of complex brain networks in neuropsychiatric disorders. Brain Connectivity.

[7] Toga AW, Clark KA, Thompson PM, Shattuck DW, Van Horn JD (2012). Mapping the human connectome. Neurosurgery.

[8] Cannataro M, Guzzi PH, Veltri P (2010). Protein-to-protein interactions: Technologies, databases, and algorithms. ACM Comput Surv (CSUR).

[9] Lesne A (2006). Complex networks: from graph theory to biology. Lett Math Phys.

[10] Bullmore E, Sporns O (2009). Complex brain networks: graph theoretical analysis of structural and functional systems. Nat Rev Neurosci.

[11] Dance A (2015). Neuroscience: Connectomes make the map. Nature.

[12] Yap PT, Wu G, Shen D (2010). Human brain connectomics: networks, techniques, and applications [life sciences]. IEEE Signal Process Mag.

[13] Tymofiyeva O, Ziv E, Barkovich AJ, Hess CP, Xu D (2014). Brain without anatomy: construction and comparison of fully network-driven structural mri connectomes. PloS ONE.

[14] Stelzl U, Worm U, Lalowski M, Haenig C, Brembeck FH, Goehler H, Stroedicke M, Zenkner M, Schoenherr A, Koeppen S (2005). A human protein-protein interaction network: a resource for annotating the proteome. Cell.

[15] Ciriello G, Mina M, Guzzi PH, Cannataro M, Guerra C (2012). AlignNemo: A Local Network Alignment Method to Integrate Homology and Topology. PloS ONE.

[16] Saraph V, Milenković T (2014). MAGNA: Maximizing accuracy in global network alignment. Bioinformatics.

[17] Neyshabur B, Khadem A, Hashemifar S, Arab SS (2013). Netal: a new graph-based method for global alignment of protein–protein interaction networks. Bioinformatics.

[18] Patro R, Kingsford C (2012). Global network alignment using multiscale spectral signatures. Bioinformatics.

[19] Ibragimov R, Malek M, Guo J, Baumbach J (2013). Gedevo: an evolutionary graph edit distance algorithm for biological network alignment. OASIcs-OpenAccess Series in Informatics.

[20] Sun Y, Crawford J, Tang J, Milenkovic T (2015). Simultaneous optimization of both node and edge conservation in network alignment via wave. International Workshop on Algorithms in Bioinformatics.

[21] El-Kebir M, Heringa J, Klau GW (2011). Lagrangian relaxation applied to sparse global network alignment. IAPR International Conference on Pattern Recognition in Bioinformatics.

[22] Thirion B, Varoquaux G, Dohmatob E, Poline JB (2014). Which fmri clustering gives good brain parcellations?. Front Neurosci.

[23] Fornito A, Zalesky A, Breakspear M (2013). Graph analysis of the human connectome: promise, progress, and pitfalls. Neuroimage.

[24] Geyer S, Weiss M, Reimann K, Lohmann G, Turner R (2011). Microstructural parcellation of the human cerebral cortex–from brodmann’s post-mortem map to in vivo mapping with high-field magnetic resonance imaging. Front Hum Neurosci.

[25] Guzzi PH, Milenkovìc T. Survey of local and global biological network alignment: the need to reconcile the two sides of the same coin,Briefings in Bioinformatics: Oxford University Press UK; 2017. Epub ahead of print. https://doi.org/10.1093/bib/bbw132. 10.1093/bib/bbw132 28062413

[26] Mina M, Guzzi PH (2014). Improving the robustness of local network alignment: design and extensive assessment of a markov clustering-based approach, IEEE/ACM Transactions on Computational Biology and Bioinformatics (TCBB),11 3.

[27] Saraph V, Milenković T (2014). Magna: maximizing accuracy in global network alignment. Bioinformatics.

[28] Crawford J, Sun Y, Milenković T (2015). Fair evaluation of global network aligners. Algorithms Mol Biol.

[29] Kuchaiev O, Pržulj N (2011). Integrative network alignment reveals large regions of global network similarity in yeast and human. Bioinformatics.

[30] Vijayan V, Saraph V, Milenković T (2015). Magna++: Maximizing accuracy in global network alignment via both node and edge conservation. Bioinformatics.

